# Development of microsatellite markers for population genetics of biting midges and a potential tool for species identification of *Culicoides sonorensis* Wirth & Jones

**DOI:** 10.1186/s13071-022-05189-8

**Published:** 2022-03-02

**Authors:** Phillip Shults, Megan Moran, Alexander J. Blumenfeld, Edward L. Vargo, Lee W. Cohnstaedt, Pierre-Andre Eyer

**Affiliations:** 1 USDA-ARS, Foreign Arthropod-Borne Animal Diseases Research Unit (FABADRU), 1515 College Ave, Manhattan, KS 66502 USA; 2 Department of Entomology, Texas A&M University, College Station, TX 77843 USA

**Keywords:** Vector surveillance, Biting midges, Molecular identification, PCR, Single-tube assay

## Abstract

**Background:**

Proper vector surveillance relies on the ability to identify species of interest accurately and efficiently, though this can be difficult in groups containing cryptic species. *Culicoides* Latreille is a genus of small biting flies responsible for the transmission of numerous pathogens to a multitude of vertebrates. Regarding pathogen transmission, the *C. variipennis* species complex is of particular interest in North America. Of the six species within this group, only *C. sonorensis* Wirth & Jones is a proven vector of bluetongue virus and epizootic hemorrhagic disease virus. Unfortunately, subtle morphological differences, cryptic species, and mitonuclear discordance make species identification in the *C. variipennis* complex challenging. Recently, single-nucleotide polymorphism (SNP) analysis enabled discrimination between the species of this group; however, this demanding approach is not practical for vector surveillance.

**Methods:**

The aim of the current study was to develop a reliable and affordable way of distinguishing between the species within the *C. variipennis* complex, especially *C. sonorensis*. Twenty-five putative microsatellite markers were identified using the *C. sonorensis* genome and tested for amplification within five species of the *C. variipennis* complex. Machine learning was then used to determine which markers best explain the genetic differentiation between species. This led to the development of a subset of four and seven markers, which were also tested for species differentiation.

**Results:**

A total of 21 microsatellite markers were successfully amplified in the species tested. Clustering analyses of all of these markers recovered the same species-level identification as the previous SNP data. Additionally, the subset of seven markers was equally capable of accurately distinguishing between the members of the *C. variipennis* complex as the 21 microsatellite markers. Finally, one microsatellite marker (*C508*) was found to be species-specific, only amplifying in the vector species *C. sonorensis* among the samples tested.

**Conclusions:**

These microsatellites provide an affordable way to distinguish between the sibling species of the *C. variipennis* complex and could lead to a better understanding of the species dynamics within this group. Additionally, after further testing, marker *C508* may allow for the identification of *C. sonorensis* with a single-tube assay, potentially providing a powerful new tool for vector surveillance in North America.

**Graphical Abstract:**

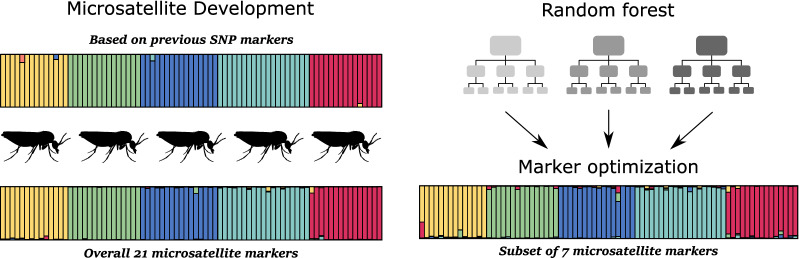

**Supplementary Information:**

The online version contains supplementary material available at 10.1186/s13071-022-05189-8.

## Background

With newly diverged or cryptic species, the boundaries between taxa can be blurred and often difficult to define [1, 2]. Yet, species delimitation is vitally important, as it determines the biological unit on which governmental policies, control programs, evolutionary studies, and conservation efforts rely [3, 4]. This is especially true for species that pose a risk to public or animal health, such as pathogen vectors, as misidentifications will result in unreliable transmission data. Morphological identification is commonly used in vector surveillance due to its wide accessibility and cost-effectiveness, though it can require a considerable amount of expertise if the target species closely resembles a sibling species or if it exhibits extensive morphological variation [5]. In many cases, sequencing a common barcoding region (i.e., cytochrome oxidase subunit 1 [COI]) can be done with far less training while providing a tangible level of taxonomic identification [6]. However, barcoding is neither easily implemented nor cost-effective for use in vector surveillance programs that process hundreds, if not thousands, of specimens. For a molecular marker to be feasible in these situations, species-specific amplification is needed, denoting either the presence or absence of the vector species within pools of samples [7].

*Culicoides* Latreille is a genus of small, biting midges that are responsible for the transmission of many pathogens affecting both wildlife and livestock worldwide [8, 9]. Viruses such as bluetongue virus (BTV) and epizootic hemorrhagic disease virus (EHDV) are of particular interest, as these can cause a high rate of mortality in infected animals [10, 11]. In the past two decades, *Culicoides* spp. have contributed to notable disease outbreaks in Australia [12], Europe [13, 14], and North America [15, 16], leading to significant morbidity, mortality, and economic loss in these regions [10, 11]. These outbreaks highlight the need for *Culicoides* vector surveillance and population management programs; however, these are complicated by the fact that several of the vector species belong to complexes of closely related species that are not easily distinguishable [17]. The inclusion of a non-vector cryptic species into vector surveillance data can artificially lower seroprevalence rates, overestimate species distributions, or even interfere with the detection of other vector species. The *C. imicola*, *C. obsoletus*, *C. pulicaris*, and *C. variipennis* complexes all play a key role in the transmission of BTV and EHDV [9, 17]; however, proper species-level identification in these groups remains challenging. Molecular tools have been developed to aid in species identification in certain groups of *Culicoides* [18–21], though cryptic diversity is often noted in *Culicoides* taxa regarded as a single species [22–24]. Additionally, there have been no molecular markers developed for the identification of *C. sonorensis*, the North American vector of BTV and EHDV.

The *C. variipennis* species complex is found throughout much of North America and comprises at least six species (*C. albertensis* Wirth & Jones, *C*. *australis* Wirth & Jones, *C. occidentalis* Wirth & Jones, *C. sonorensis* Wirth & Jones, *C. variipennis* (Coquillett), and *C. mullensi* Shults & Borkent) [25], only one of which (*C. sonorensis*) is a proven vector [26–29]. Species delimitation within the *C. variipennis* complex is particularly challenging due to subtle morphological differences between these species [30]. Species identification is further hampered by a lack of segregation between mitochondrial haplotypes of three of these species, including the vector species *C. sonorensis* (plus *C. albertensis* and *C. variipennis*) [31]. The absence of mitochondrial discrimination prevents genetic identification using the traditional COI barcode [32–34], and to further complicate the situation, *C. sonorensis* occurs in sympatry with each of the other members of this species complex [35]. Overall, the lack of clear morphological differences, the unavailability of readily applied genetic identification, and the occurrence of several species within a single location have introduced ambiguity to vector surveillance in this group. Recently, genomic analyses using a single-nucleotide polymorphism (SNP) dataset shed light on species delimitation in the *C. variipennis* complex and served as a useful tool for population genomic analyses [31]. However, this method is expensive and requires bioinformatics analyses, rendering it unsuitable for the rapid and affordable species identification necessary for effective vector surveillance.

Here, we first aimed to provide an easy and cost-effective way to identify species within the *C. variipennis* complex, especially the vector species *C. sonorensis*. We developed microsatellite markers from the available genome of *C. sonorensis* and tested these markers’ ability to distinguish between the species within the *C. variipennis* complex. These results were compared to the species delimitation obtained through SNP analyses from Shults et al. [31]. We then used machine-learning analyses to estimate the influence of each microsatellite marker in discriminating between the different species in the *C. variipennis* complex. This analysis was used to determine the minimum number of markers required for identification while still maintaining a high level of confidence. Finally, a single marker was found to uniquely amplify in the *C. sonorensis* samples tested and may prove to be a fast and inexpensive tool for discriminating between this species and the non-vector species of the *C*. *variipennis* complex.

## Methods

### Microsatellite marker selection

The reference genome of *C. sonorensis* (RefSeq GCA_900258525.2) [36] was assessed with the QDD v.3.1 software program [37] to determine suitable microsatellite repeat motifs. Microsatellite repeats containing less than five repetitions, as well as mononucleotide repeats, were discarded. For each microsatellite repeat, 200-base-pair (bp) flanking regions on either side of the repeat were extracted. Overall, microsatellite repeat motifs were identified in 60,026 reads. To maximize polymorphism, loci with the highest number of repeats were selected, all of which had dinucleotide repeats. Twenty-five loci were selected, and their corresponding primers were generated using the online Primer-BLAST software through NCBI (https://www.ncbi.nlm.nih.gov/tools/primer-blast). A broad range of polymerase chain reaction (PCR) products (110–490) were targeted to facilitate the development of multiplex arrangements. Primer sequences, microsatellite repeat information, and product size are displayed for each of the microsatellite markers in Table 1.

**Table 1:** List of the primers used to amplify each microsatellite (MS) marker

Marker	Primer sequence	MS motif	No. of repeats	Product size (bp)
*C1241*	F: CACGACGTTGTAAAACGACACAACAAACAACGGAGTCATGTTTA	TG	35	203–277
	R: TAGCTCACATACGTTAAATGTCAAA			
*C65*	F: CACGACGTTGTAAAACGACACACGCGATTATGCATTTTGTAGT	AC	29	143–236
	R: TTATCGTTGCAACCCATCATTA			
*C927*	F: CACGACGTTGTAAAACGACACTTCACGCATGAATGCACATAA	TG	41	201–225
	R: TTCGGTTGGAAACTGATACACA			
*C226*	F: CACGACGTTGTAAAACGACACTGCAACAGAATCAATTCCATGA	TG	45	149–238
	R: TGTTTGAAGCAACAGAAGCG			
*C2085*	F: CACGACGTTGTAAAACGACACTGCTTGATTTTCTGCCAACTCA	AC	57	171–223
	R: GCATCAACAACATTTGTATATCGCA			
*C47*	F: CACGACGTTGTAAAACGACACTGTCAAACCAGATTGAGCCA	AC	26	152–203
	R: TGATGATCACACACGATAACCA			
*C43*	F: CACGACGTTGTAAAACGACACACAGGAAACACCCTTACAAAAC	TG	32	307–360
	R: TGCAAATGCGACTCTTGATT			
*C244*	F: CACGACGTTGTAAAACGACACACTTTTCAATTCAAGCTGCTACT	GT	31	118–161
	R: TTCGTTCATTGTGCATTTCATT			
*C838*	F: CACGACGTTGTAAAACGACACGTTGCGATGCAACACATGA	TG	32	425–487
	R: ACAATAAAGCAACAACAAGGGT			
*C230*	F: CACGACGTTGTAAAACGACACTTTTCCCAATCACCCTGGA	CA	33	127–250
	R: CAGCTAGAACAACAGTGAAAGG			
*C589*	F: CACGACGTTGTAAAACGACACACTCTGGATAATTGGTGTCACG	TG	26	161–221
	R: CCACACAAATCAATGCCCCT			
*C54*	F: CACGACGTTGTAAAACGACACCAAGACTTTTAAGTGTAACCACACA	AC	26	117–168
	R: CCAACAGCGTCATATCATCTTACAT			
*C1450*	F: CACGACGTTGTAAAACGACACTCCAAAGTACCATGACCGTCT	AC	26	214–274
	R: GGAGAAACACACGAACCCTT			
*C424*	F: CACGACGTTGTAAAACGACACTTGTGTGTTGTTGAGGGTTCA	GT	47	253–284
	R: GAAGTTCGTCAAGGTCAAGCA			
*C94*	F: CACGACGTTGTAAAACGACACTGGTCTCATACGACCCATTAACA	GT	27	176–230
	R: TCAGAGTGTTTGCAGAGATGC			
*C1296*	F: CACGACGTTGTAAAACGACACATCACAATGGACAAGTATGTCG	GT	30	135–222
	R: AATCATGAGACAGGACCAAGA			
*C1253*	F: CACGACGTTGTAAAACGACACGATCGACAACAGACGACTCAT	GT	29	206–270
	R: TCTCTCTCTGCTTGGTTTCTATT			
*C995*	F: CACGACGTTGTAAAACGACACTCCTGGAAAATCTAATAAGGCAA	CA	28	326–394
	R: GTACATTGTGTATTTGTACCAAGTT			
*C508*	F: CACGACGTTGTAAAACGACACTGCCTCATGCAAACTCTCTTC	AC	30	337–364
	R: GAAGATGTATAGCAAAATGGGTGA			
*C728*	F: CACGACGTTGTAAAACGACACGCACCAGCAATTTTCTGTCT	TG	40	433–484
	R: ACGCAACATTTGGTGTAGTG			
*C45*	F: CACGACGTTGTAAAACGACACGTCGCAAGGTAGGTCATTTTTC	AC	38	406–460
	R: TGTGTCGATCTGTGAAACATCT			

### Molecular techniques

**Fig. 1:**
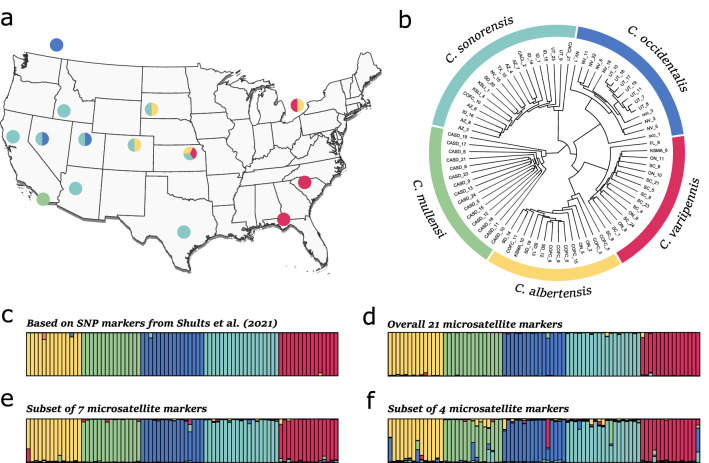
**a** The sites used to collect specimens of the *C. variipennis* complex. Each location is colored (corresponding to the phylogenetic tree) to represent which species were tested from these collection sites. corresponding to the phylogenetic tree. **b** A phylogenetic tree and **c** STRUCTURE plot based on SNP data from the subset of individuals sequenced in Shults et al. [31] (best *K* = 5). Structure plots inferred from **d** the 21-marker dataset (best *K* = 5), the seven-marker dataset (best *K* = 5) (**e**), and the four-marker dataset (best *K* = 5) (**f**)

A total of 79 individuals from five selected species of the *C. variipennis* complex (14 for *C. albertensis,* 15 for *C. mullensi,* 16 for *C. occidentalis,* 19 for *C. sonorensis*, and 15 for *C. variipennis;* Additional file 4: Table S1) were used for testing primer amplification. These individuals were collected using Centers for Disease Control and Prevention (CDC) light traps in both rural and semi-urban areas during previous study. Individuals were assigned to species based on genomic analyses of 3609 SNP loci from Shults et al. [31] and were selected for the current study to ensure coverage of most of the geographic distributions of the different species (Fig. 1a, b). A modified Gentra Puregene extraction method (Gentra Systems, Inc. Minneapolis, MN, USA) was used to extract the genomic DNA of each individual. Each of the 25 primers was amplified in standard simplex PCR conditions using a Bio-Rad T100 thermal cycler (Bio-Rad, Pleasanton, CA, USA). Each PCR reaction contained 2.0 μl of DNA, 0.75 μM of a primer pair, 5.0 μl of 5 × reaction buffer, 0.15 μl of Taq, and 16.35 μl of deionized water. The cycling conditions used for the amplification of microsatellite markers consisted of 95 °C for 3 min, followed by 35 cycles of 95 °C for 1 min, 57 °C for 1.5 min, and 72 °C for 2 min, with a final extension step at 72 °C for 5 min. All microsatellite markers were tested at 57 °C, regardless of their species of origin. The M13-tailed primer method was used to label amplicons to facilitate multiplexing after PCR amplification. Each forward primer had an M13 tail attached, which was 5′-fluorescently labeled with 6-NED, VIC, PET, or FAM. An ABI 3500 capillary sequencer with a LIZ500 internal standard (Applied Biosystems, Foster City, CA, USA) was used to visualize PCR products. Alleles were scored using Geneious v.9.1 software (Biomatters, Auckland, New Zealand) [38]. Four primers were discarded due to inconsistent or nonexistent amplification. The final primer set includes 21 microsatellite markers grouped in five different multiplexes (Table 1).

### Allelic diversity and summary statistics for each species

For each marker, the number of alleles and allelic frequency were calculated for each species as well as the entire dataset using GENEPOP on the web [39]. This software was also used to calculate linkage disequilibrium on the entire dataset between each pair of microsatellite markers. GENEPOP on the web was also used separately on each species dataset to calculate the expected (H_e_) and observed (H_o_) heterozygosity, the occurrence of a significant deviation from Hardy–Weinberg equilibrium (HWE), and the inbreeding coefficient (*F*_IS_). H_e_, H_o_, deviation from HWE and *F*_IS_ were calculated separately for each marker.

### Assessing genetic differentiation of the microsatellite markers

Species-level divergence using all 21 microsatellite markers was first visualized by plotting individuals on a principal component analysis (PCA) using the *adegenet* R package [40]. A clear delimitation between species is denoted by a non-overlap of individuals from different species on the PCA. Bayesian clustering implemented in STRUCTURE v.2.3.4 [41] was used to estimate the number of genetic clusters (*K*) and determine whether individuals from distinct species cluster together. Simulations were run with values of *K* from 1 to 20 and repeated 10 times for each *K*-value. Each run consisted of a 5 × 10^4^ burn-in period followed by 1 × 10^5^ iterations of the Markov chain Monte Carlo (MCMC) algorithm. The most likely number of genetic clusters (*K*) was inferred using the method described by Puechmaille [42] implemented in StructureSelector web-based software [43]. The outputs were visualized using CLUMPAK [44]. The clustering of individuals from the different species using the microsatellite datasets was compared to the clustering of the same individuals using the SNP dataset from Shults et al. [31] (Fig. 1c, Additional file 6: SNP dataset available at https://doi.org/10.17605/OSF.IO/E3Z72).

### Selecting a subset of markers for optimized species differentiation

A random forest (RF) classification analysis was carried out on an 18-microsatellite dataset using the R package *randomForest* [45]. Three markers (*C424*, *C995*, and *C508*) were discarded due to the presence of missing data (e.g., non-amplifying marker in some species), which cannot be handled by an RF analysis. This analysis aims at estimating the confidence rate in determining an individual’s species of origin for each of the microsatellite markers developed. The RF analysis was performed using 1000 trees. The default values were used for the number of input variables randomly selected to build each node of the tree, and for the number of observations not used for building the tree (i.e., the out-of-bag [OOB] sample). The OOB samples were used to build the confusion matrix and to estimate the OOB error rate. Low OOB error rates indicate a high ability of the variables in predicting the species of origin of the individual. In addition, RF analysis on the 18-marker dataset was used to determine the importance of each microsatellite marker in classifying the individuals in the five species. This analysis enables the selection of a subset of microsatellite markers most capable of distinguishing between species.

The markers determined to have the highest influence in separating species were grouped into two subsets (a four- and seven-marker dataset), from which a PCA and STRUCTURE analysis were subsequently applied. STRUCTURE assignments using the four- and seven-marker datasets were compared to the assignments from the entire 21 microsatellite marker dataset as well as the SNP dataset. In addition, RF analyses were re-applied on these datasets to estimate the confidence (i.e., OOB error rate) in estimating an individual’s species of origin using only four or seven microsatellite markers.

## Results

The 21 selected microsatellite markers amplified in most of the species of the *C. variipennis* complex, with a few exceptions (Table 3.) All 21 markers were found to be polymorphic, with the number of alleles ranging from 11 to 37 (mean ± SD = 26.4 ± 7.4; Table 2). More specifically, allelic diversity ranged from 3 to 15 (mean ± SD = 8.6 ± 3.4) alleles per marker for *C. albertensis*, 4 to 12 (8.4 ± 3.2) for *C. mullensi*, 4 to 16 (8.0 ± 3.9) for *C. occidentalis*, 4 to 20 (13.0 ± 3.8) for *C. sonorensis*, and 4 to 14 (8.6 ± 3.7) for *C. variipennis*. Deviation from HWE was observed for most markers per species. This result originated from significantly positive *F*_IS_ inbreeding coefficients observed for the majority of the markers and most species, with levels of observed heterozygosity lower than expected (Table 3). It is important to note that the positive *F*_IS_ values can be overestimated due to the sampling of a few individuals per species over an expansive range (i.e., the Wahlund effect). Results from the linkage disequilibrium analysis suggest that most genotypes at one locus were independent from genotypes at another locus. An exception was that markers *C45*, *C728*, and *C995* appeared to be linked (*P* = 0.004, 0.03 and 0.058), as were markers *C94* and *C2085* (*P* = 0.05) (Additional file 5: Table S2). Note that only a single marker from each of these two groups was later used in the four- and seven-marker datasets.

**Table 2:** Allelic diversity of each marker by species

Marker	Number of alleles					
	Overall	*C. albertensis*	*C. mullensi*	*C. occidentalis*	*C. sonorensis*	*C. variipennis*
*C1241*	37	13	11	9	14	11
*C65*	34	11	12	13	14	9
*C927*	16	6	5	4	10	6
*C226*	31	3	10	6	16	4
*C2085*	25	11	8	7	12	7
*C47*	28	8	9	8	15	12
*C43*	28	8	10	12	13	11
*C244*	17	6	7	9	8	8
*C838*	36	8	11	12	20	10
*C230*	27	9	4	6	19	14
*C589*	35	15	11	7	15	8
*C54*	19	9	6	10	6	11
*C1450*	31	11	12	10	17	9
*C424*	15	4	4	4	4	5
*C94*	29	12	10	10	11	5
*C1296*	35	10	10	16	16	10
*C1253*	24	9	11	7	12	8
*C995*	20	7	5	0	11	4
*C508*	11	0	0	0	11	0
*C728*	32	10	10	7	15	15
*C45*	25	11	10	12	13	14
*Mean*	26.4	8.6	8.4	8.0	13.0	8.6
*SD*	7.4	3.4	3.2	3.9	3.8	3.7

**Table 3:** The summary statistics of each marker grouped by species

Marker	*C. albertensis*				*C. mullensi*				*C. occidentalis*				*C. sonorensis*				*C. variipennis*			
	H_e_	H_o_	*F*_IS_	HWE	H_e_	H_o_	*F*_IS_	HWE	H_e_	H_o_	*F*_IS_	HWE	H_e_	H_o_	*F*_IS_	HWE	H_e_	H_o_	*F*_IS_	HWE
*C1241*	0.92	0.43	0.54	***	0.81	0.53	0.35	*	0.85	0.88	−0.03	***	0.91	0.37	0.60	***	0.86	0.60	0.31	***
*C65*	0.88	0.50	0.44	***	0.91	0.80	0.12	NS	0.88	0.63	0.29	***	0.92	0.63	0.32	***	0.90	0.33	0.64	***
*C927*	0.76	0.60	0.22	NS	0.66	0.46	0.31	NS	0.73	0.50	0.33	**	0.83	0.12	0.86	***	0.85	0.00	1.00	***
*C226*	0.36	0.43	−0.19	NS	0.77	0.73	0.04	NS	0.76	0.56	0.27	*	0.93	0.37	0.61	***	0.66	0.67	0.00	NS
*C2085*	0.90	0.79	0.13	NS	0.80	0.53	0.35	**	0.66	0.38	0.44	**	0.88	0.47	0.47	***	0.81	0.60	0.27	NS
*C47*	0.80	0.64	0.20	NS	0.84	0.73	0.13	NS	0.72	0.69	0.04	NS	0.91	0.53	0.43	***	0.87	0.67	0.24	***
*C43*	0.77	0.79	−0.02	NS	0.85	0.87	−0.03	NS	0.90	0.44	0.52	***	0.87	0.58	0.34	**	0.93	0.86	0.08	NS
*C244*	0.71	0.21	0.71	***	0.77	0.33	0.58	***	0.87	0.44	0.51	***	0.84	0.22	0.74	***	0.93	0.43	0.55	***
*C838*	0.73	0.36	0.52	**	0.87	0.64	0.27	*	0.88	0.56	0.36	***	0.95	0.84	0.12	NS	0.82	0.67	0.19	NS
*C230*	0.85	0.29	0.67	***	0.36	0.33	0.07	NS	0.72	0.31	0.58	***	0.95	0.68	0.29	**	0.87	0.67	0.24	***
*C589*	0.92	1.00	−0.10	NS	0.85	0.53	0.38	*	0.73	0.44	0.41	*	0.90	0.74	0.18	*	0.78	0.73	0.06	NS
*C54*	0.85	0.71	0.17	NS	0.79	0.80	−0.01	NS	0.86	0.69	0.21	*	0.73	0.47	0.36	**	0.83	0.67	0.20	*
*C1450*	0.87	0.79	0.10	NS	0.89	0.87	0.03	NS	0.89	0.56	0.37	*	0.94	0.63	0.33	***	0.82	0.87	−0.06	NS
*C424*	0.27	0.07	0.74	**	0.75	0.25	0.70	*	0.49	0.10	0.80	***	0.80	0.00	1.00	**	0.31	0.27	0.14	NS
*C94*	0.87	0.79	0.10	NS	0.86	0.73	0.15	NS	0.79	0.53	0.34	*	0.87	0.68	0.22	*	0.62	0.47	0.25	NS
*C1296*	0.89	0.64	0.29	NS	0.90	0.67	0.26	*	0.93	0.63	0.34	***	0.90	0.68	0.25	*	0.86	0.80	0.08	NS
*C1253*	0.84	0.71	0.16	**	0.90	0.27	0.71	***	0.77	0.38	0.51	***	0.88	0.65	0.27	NS	0.85	0.53	0.38	**
*C995*	0.83	0.20	0.77	***	0.77	0.38	0.53	*	-	-	-	-	0.93	0.08	0.92	***	0.65	0.27	0.59	*
*C508*	-	-	-	-	-	-	-	-	-	-	-	-	0.90	0.32	0.66	***	-	-	-	-
*C728*	0.89	0.93	−0.05	NS	0.87	0.87	0.00	NS	0.85	0.62	0.29	**	0.92	0.72	0.22	*	0.93	1.00	−0.08	NS
*C45*	0.89	0.62	0.32	***	0.89	0.64	0.28	**	0.92	0.56	0.39	***	0.90	0.74	0.18	*	0.92	0.80	0.13	***
Overall	0.79	0.57	0.29		0.81	0.60	0.26		0.80	0.52	0.37		0.89	0.50	0.45		0.80	0.59	0.26	

**Fig. 2:**
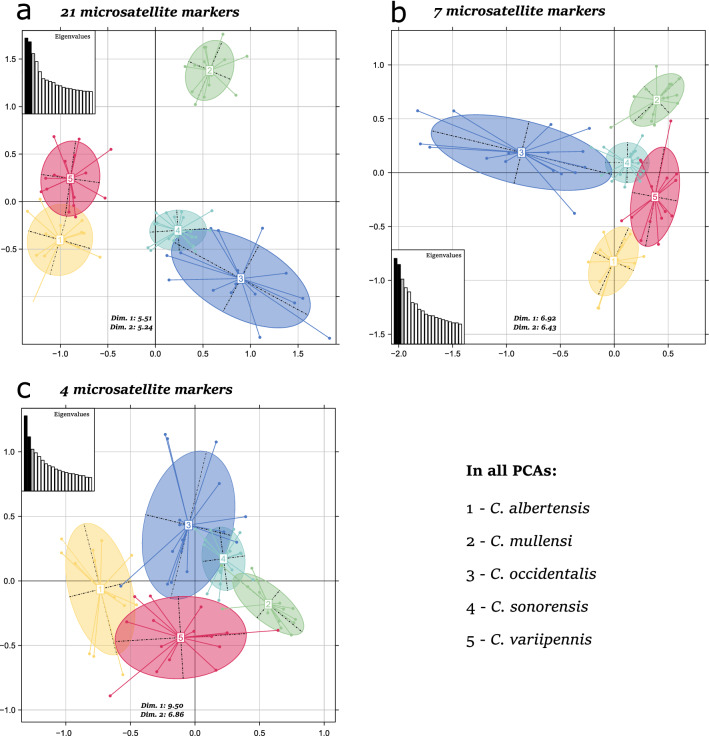
Principal component analysis (PCA) of individuals of the *C. variipennis* complex using the **a** 21-marker, **b** seven-marker, and **c** four-marker datasets. Each dot represents an individual and each color corresponds to the species assignment generated from the STRUCTURE analyses. The shaded ovals denote the confidence ellipse for the mean of each species

The overall dataset of 21 markers was successful in the species-level differentiation of all specimens, though the clustering of individuals using a PCA revealed that two species, *C.* *albertensis* and *C.* *variipennis*, overlap slightly (Fig. 2a). The clustering of individuals using a STRUCTURE analysis suggested the presence of five distinct clusters in the dataset (best *K* = 5; Fig. 1d; individual assignments for other values of *K* are provided in Additional file 1: Figure S1). This clustering using microsatellite markers corresponds to five different species, as it closely mirrors the results of the SNP dataset with the same samples from Shults et al. [31] (Fig. 1c). Importantly, individuals mostly belonged (> 85% [mean = 98%] assignment probability) to a single genetic cluster when using the overall dataset of 21 microsatellite markers (i.e., unambiguous assignment to the correct species). RF analysis on the overall dataset also suggests that markers *C226*, *C728*, *C838*, and *C1450* had the highest influence in distinguishing between species, followed by markers *C589*, *C2085*, and *C1241* (Additional file 2: Figure S2). When using most of the microsatellite markers (18-marker dataset), the OOB error rate was 1.3%. The confusion matrix found that a potential low rate of misidentifications might occur with *C. sonorensis* and *C.* *variipennis* samples, while no misidentification occurs among samples from the three other species (Additional file 3: Figure S3).

When the seven-marker dataset was analyzed (i.e., *C226*, *C728*, *C838*, *C1450*, *C589*, *C2085*, and *C1241*), almost no overlap was found between individuals from distinct species on the PCA (Fig. 2b). Similarly, STRUCTURE analysis revealed confident segregation of the individuals into the different species (Fig. 1e), as most individuals (*N* = 75) were unambiguously assigned to the correct species (> 85% [mean = 95%] assignment probability). Only four samples had assignment probabilities lower than 85% to the correct species cluster, with one sample of *C. occidentalis* (63%), one sample of *C. albertensis* (71%), and two samples of *C. variipennis* (80 and 83%). Additionally, the clustering closely mirrored the results from both the entire 21 microsatellite marker dataset and the SNP dataset. This finding suggests robust segregation of samples into the different species using seven microsatellite markers. The RF analysis provides further support for species delineation using these markers, revealing an OOB error rate of 1.9% (Additional file 3: Figure S3). The confusion matrix found a misclassified sample of *C. mullensi*, *C. sonorensis*, and *C. variipennis*.

When plotting individuals on a PCA using the four-marker dataset (i.e., *C226*, *C728*, *C838*, and *C1450*), individuals within the same species mostly clustered together, despite small overlap (Fig. 2c). Similarly, the STRUCTURE analysis revealed that individuals mostly cluster into their respective species (Fig. 1f), with most individuals (*N* = 65) being correctly assigned (> 85% [mean = 87%] assignment probability). However, 14 individuals had a mixed assignment (< 85% assignment probability), with four of them having less than 50% assignment to their correct species, hampering full confidence in identifying species using only four markers. This finding was confirmed by an RF analysis that revealed a small, but non-negligible OOB error rate of 6.3% (Additional file 3: Figure S3). The confusion matrix revealed multiple misclassified samples belonging to *C. mullensi, C. sonorensis*, and *C. variipennis*.

**Fig. 3:**
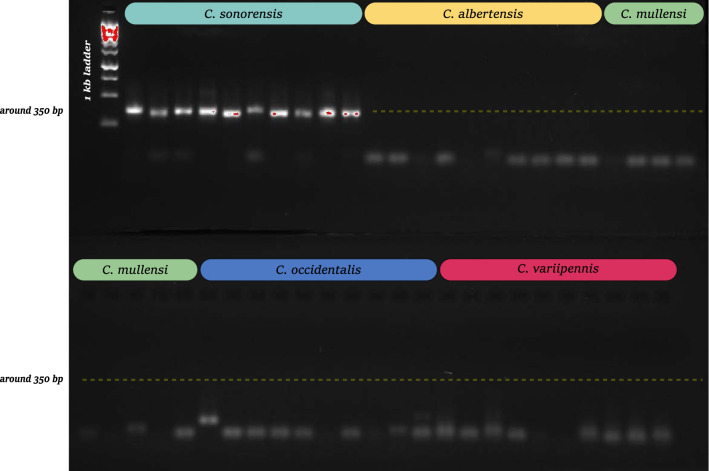
The PCR product of marker *C508* imaged after gel electrophoresis for individuals of the *C. variipennis* complex. This marker is roughly 350 bp in length and, of the samples tested, shows amplification in only *C. sonorensis*. These specimens represent a subset of individuals tested in this study. For each species, at least one individual from each sample location (Fig. 1a) is shown here

Lastly, the microsatellite locus *C508* was found to amplify only in *C. sonorensis* (Fig. 3), the only proven vector species within the *C. variipennis* complex. In total, 79 individuals spanning 14 geographic locations were tested at this marker: *C. albertensis* from four populations, *C. mullensi* from one population, *C. occidentalis* from three populations, *C. sonorensis* from nine populations, and *C. variipennis* from four populations (Fig. 1a and Additional file 4: Table S1). Many more samples and populations need to be tested to confirm this species-specific amplification; however, in the samples tested here, there does not appear to be any geographical bias in amplification. Individuals of *C. albertensis*, *C. occidentalis*, and *C. variipennis* collected from the same location as individuals of *C. sonorensis* showed no amplification at this marker. It is also important to note that this marker was not included in the RF analyses above due to the substantial amount of missing data (i.e., non-amplification in four of the sibling species) (Table 3).

## Discussion

As only about 2% of the known species of *Culicoides* are vectors [8], differentiating these from non-vector species remains vital to surveillance efforts. Since this can be complicated by morphologically similar cryptic taxa, molecular species delimitation tools are needed. This study identified a relatively simple, reproducible, and economical tool for the molecular differentiation of the species within the *C. variipennis* species complex. We generated a set of 21 microsatellite markers that can assign species-level identities to the members of this complex. These markers also exhibit consistent polymorphism for each species and should lead to a better understanding of the population structure and species dynamics within this group. Machine learning was utilized to detect a set of seven microsatellite markers optimal for distinguishing between these species, further reducing costs. Finally, the locus *C508* was found to only amplify in *C. sonorensis* and appears to be a promising marker to improve vector surveillance for this species, though additional testing is needed.

In populations with closely related or cryptic species, approaching species delimitation at a population level can help identify independent, or mostly independent, gene pools. Shults et al. [31] provided insight into the number of biological species within the *C. variipennis* complex; however, SNP data is expensive to produce and cannot be easily combined with new datasets. Conversely, the microsatellite data produced here was far less expensive while achieving the same level of species delimitation as the SNPs. This will allow these new markers to be integrated into most studies of this species complex to improve accurate species identification. It is highly likely that the species distribution records and serological data within this group need to be revisited. Additionally, as morphological identification of the larvae within the *C. variipennis* complex is not possible, these markers will help to decipher the immature habitat of each species. While not common in nature, hybridization is possible between *C. sonorensis* and both *C. occidentalis* and *C. variipennis* [31, 46]. Because these three species are well separated using both 21-marker and 7-marker datasets, it is likely that these microsatellites datasets can be used to identify hybrid individuals (at least F1 and F2). These would have a mixed assignment in a STRUCTURE plot and fall between two clusters in a PCA. Unfortunately, this study was unable to obtain specimens of the newly elevated *C. australis*; however, if this is truly a valid species, these markers should differentiate it as well. Occurring sympatrically with *C. sonorensis* and *C. variipennis* in the southeastern USA, the main evidence for the species-level designation of *C. australis* is a differing larval habitat and subtle morphological variation on the antennae [25]. Genetic differentiation at these microsatellite markers would provide strong evidence for the validity of this species.

While these microsatellite markers will be helpful to future studies of the *C. variipennis* complex, their practicality in vector surveillance may be limited. However, locus *C508* could be incredibly useful for the rapid identification of the vector species, *C. sonorensis*. If amplification of this marker is specific to this species, screening individuals or pools of individuals can be completed with a single PCR and agarose gel. Of the samples tested here, amplification was 100% for *C. sonorensis*, which included individuals from nine populations across its known range (Additional file 4: Table S1). Conversely, no amplification was seen in the other members of the *C. variipennis* complex (Fig. 3). More samples need to be tested to be confident in locus *C508*’s ability to identify *C. sonorensis* in all populations. Additionally, this marker has yet to be tested on *Culicoides* species outside of this complex. Should this marker be cross-reactive with another species, it would produce a false positive if used as the sole method for identifying *C. sonorensis*. Fortunately, the *C. variipennis* complex is morphologically distinguishable from other species of *Culicoides* [25, 47], thus if this were the case, a combination of the two methods would still allow for rapid identification of the vector species. Single-tube molecular identification assays already exist for vector species in other *Culicoides* species complexes; however, most of these are based on mitochondrial data. If similar patterns of mitonuclear discordance (as seen in the *C. variipennis* complex) exist in these groups, these assays have the potential to miss cryptic species. As microsatellite markers have already been developed for several of these groups [48, 49], it would be interesting to compare the number of species recovered between these mitochondrial and nuclear markers.

## Supplementary Information

**Additional file 1: Figure S1.** STRUCTURE results assuming four, five, and six clusters (*K* = 4, *K* = 5, and *K* = 6). Each column represents an individual and all samples are grouped by species.

**Additional file 2: Figure S2.** Variable importance plot of each marker’s ability to categorize samples into the distinct species. The higher the value of the mean decrease in the Gini score, the higher the accuracy of species delimitation within the *C. variipennis* complex.

**Additional file 3: Figure S3.** Confusion matrices from RF analyses indicating the accuracy of each microsatellite dataset in predicting an individual’s species of origin. Mismatches between the actual species and the predicted species assignment of an individual are shown outside of the darkened diagonal. The species-specific OOB error rate is shown on the right side of each matrix. The overall OOB estimate of error roughly corresponds to the confidence interval when using these marker sets for species assignment (i.e., 18-marker = 99%, seven-marker = 98%, and four-marker = 94%).

**Additional file 4: Table S1.** The collection sites of samples used in this study. The sample names of each individual are from Shults et al. [31].

**Additional file 5: Table S2.** Genotypic linkage disequilibrium. A log-likelihood ratio statistic (G-test) was used to determine whether genotypes at one locus were independent from genotypes at another. *P*-values between pairs < 0.05 indicate statistically significant linkage between markers.

**Additional file 6.** SNP analyses.

## Data Availability

All data generated or analyzed during this study are included in this published article and its additional information files.

## Abbreviations

BTV: Bluetongue virus
COI: Cytochrome oxidase subunit 1
EHDV: Epizootic hemorrhagic disease
*F*_IS_: Inbreeding coefficient
H_e_: Expected heterozygosity
H_o_: Observed heterozygosity
HWE: Hardy–Weinberg equilibrium
OOB: Out-of-bag
PCA: Principal component analysis
PCR: Polymerase chain reaction
RF: Random forest
USA: United States of America
