# Supplementary figures and images for: Development of microsatellite markers for population genetics of biting midges and a potential tool for species identification of Culicoides sonorensis Wirth & Jones
Source: Parasit Vectors. 2022 Mar 2;15:69. doi: 10.1186/s13071-022-05189-8 (PMC8889724; doi:10.1186/s13071-022-05189-8)

## Overall 21 markers

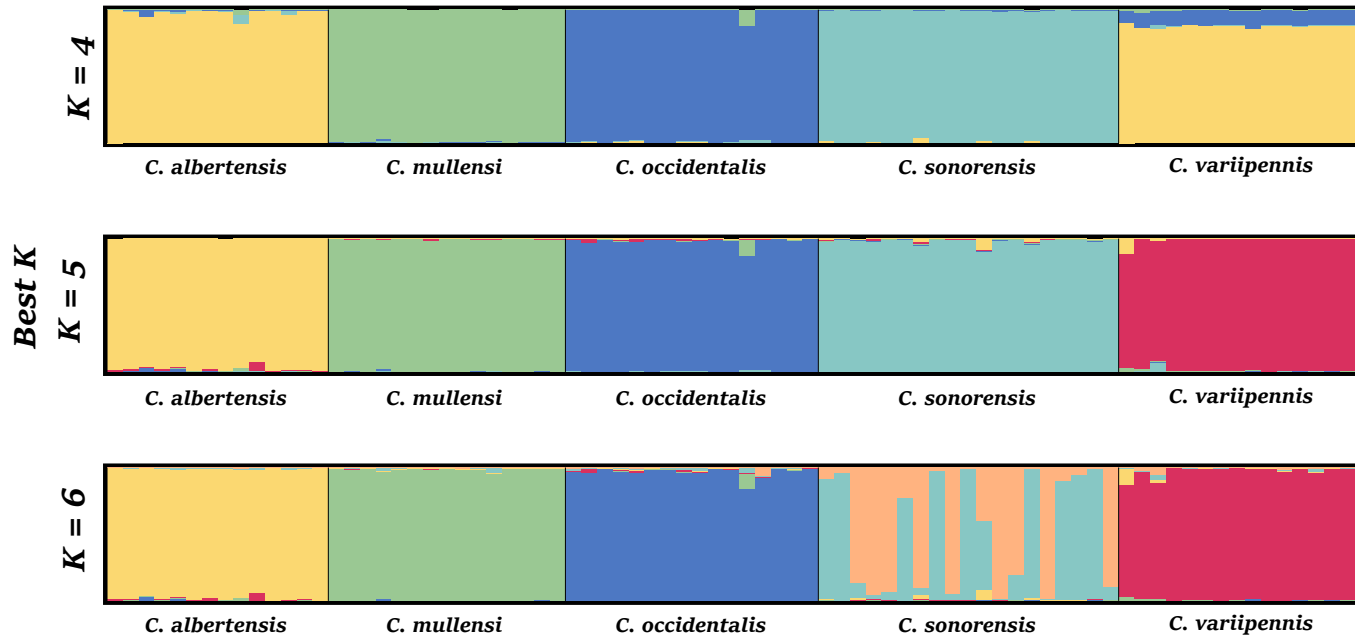

Supplement: Supplementary file 1 — Additional file 1: Figure S1. STRUCTURE results assuming four, five, and six clusters (K = 4, K = 5, and K = 6). Each column represents an individual and all samples are grouped by species. [file 13071_2022_5189_MOESM1_ESM.pdf]

**Microsatellite markers**

**4-marker subset**

**7-marker subset**

C226  
C728  
C838  
C1450  
C589  
C2085  
C1241  
C244  
C47  
C230  
C94  
C1253  
C43  
C1296  
C927  
C45  
C65  
C54

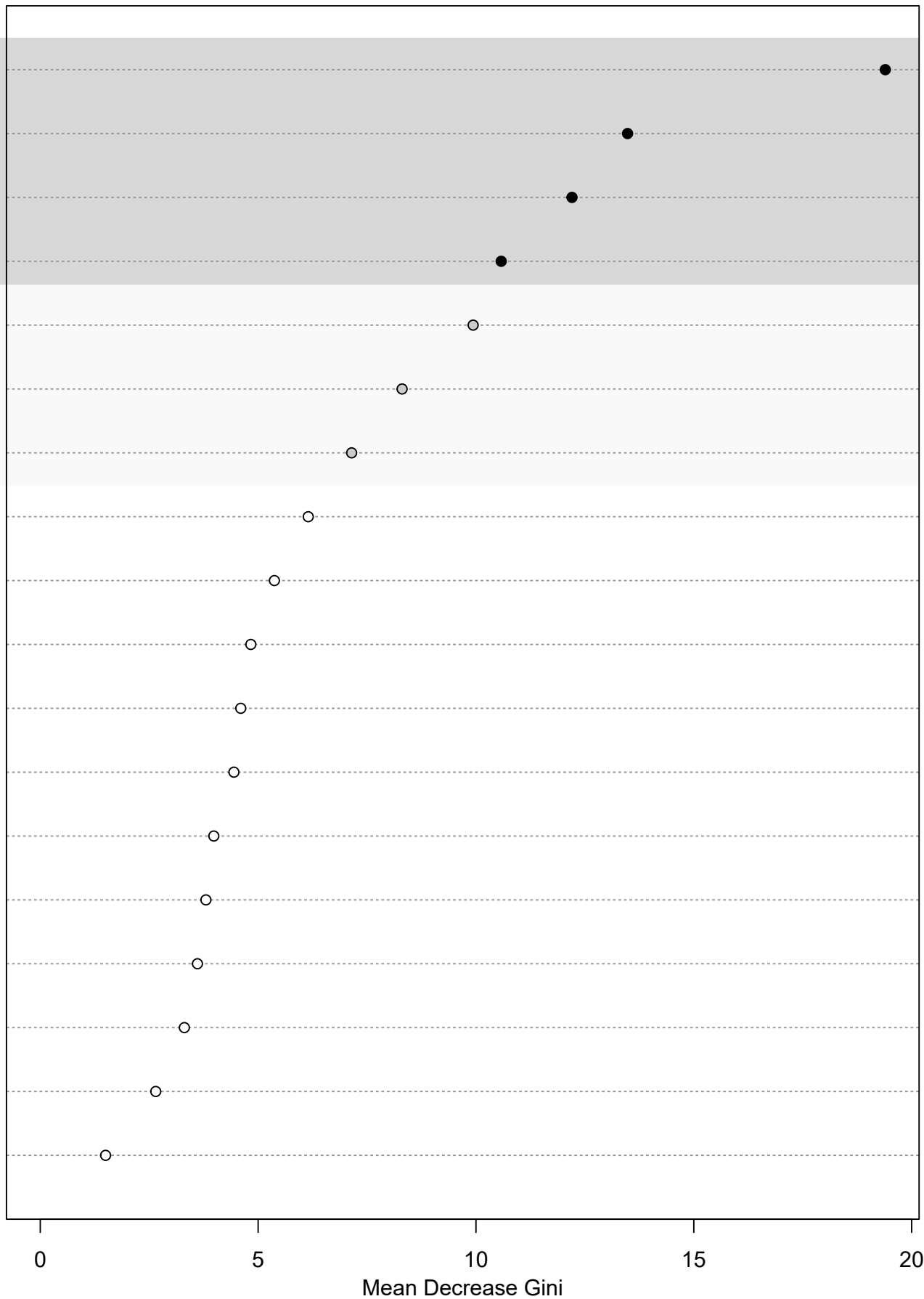

Supplement: Supplementary file 2 — Additional file 2: Figure S2. Variable importance plot of each marker’s ability to categorize samples into the distinct species. The higher the value of the mean decrease in the Gini score, the higher the accuracy of species delimitation within the C. variipennis complex. [file 13071_2022_5189_MOESM2_ESM.pdf]
