# Supplementary material for: Development of microsatellite markers for population genetics of biting midges and a potential tool for species identification of Culicoides sonorensis Wirth & Jones
Source: Parasit Vectors. 2022 Mar 2;15:69. doi: 10.1186/s13071-022-05189-8 (PMC8889724; doi:10.1186/s13071-022-05189-8)
Supplement: Supplementary file 3 — Additional file 3: Figure S3. Confusion matrices from RF analyses indicating the accuracy of each microsatellite dataset in predicting an individual’s species of origin. Mismatches between the actual species and the predicted species assignment of an individual are shown outside of the darkened diagonal. The species-specific OOB error rate is shown on the right side of each matrix. The overall OOB estimate of error roughly corresponds to the confidence interval when using these marker sets for species assignment (i.e., 18-marker = 99%, seven-marker = 98%, and four-marker = 94%). [file 13071_2022_5189_MOESM3_ESM.pdf]

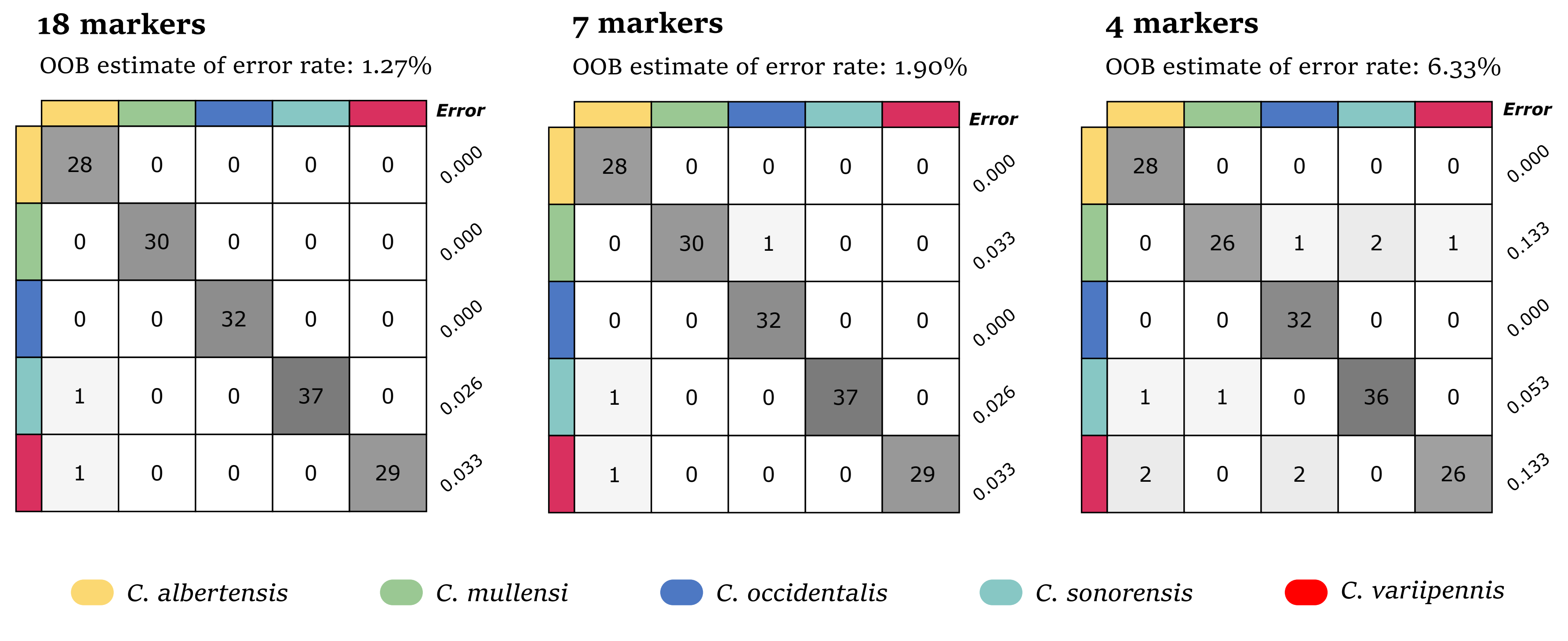
18 markers
OOB estimate of error rate: 1.27%
Error
28 0 0 0 0 0.000
0 30 0 0 0 0.000
0 0 32 0 0 0.000
1 0 0 37 0 0.026
1 0 0 0 29 0.033
7 markers
OOB estimate of error rate: 1.90%
Error
28 0 0 0 0 0.000
0 30 1 0 0 0.033
0 0 32 0 0 0.000
1 0 0 37 0 0.026
1 0 0 0 29 0.033
4 markers
OOB estimate of error rate: 6.33%
Error
28 0 0 0 0 0.000
0 26 1 2 1 0.133
0 0 32 0 0 0.000
1 1 0 36 0 0.053
2 0 2 0 26 0.133
C. albertensis
C. mullensi
C. occidentalis
C. sonorensis
C. variipennis
