# Supplementary material for: Development of microsatellite markers for population genetics of biting midges and a potential tool for species identification of Culicoides sonorensis Wirth & Jones
Source: Parasit Vectors. 2022 Mar 2;15:69. doi: 10.1186/s13071-022-05189-8 (PMC8889724; doi:10.1186/s13071-022-05189-8)
Supplement: Supplementary file 6 — Additional file 6. SNP analyses. [file 13071_2022_5189_MOESM6_ESM.docx]

**Supplemental Information**

*SNP analyses*

Shults *et*. *al*. (2021) obtained single-end 100 bp reads through ddRadseq on a HiSeq4000. This dataset was trimmed to only include individuals also included in the present study (Table S1) and raw reads from these individuals were filtered and processed using Stacks v.2.3. Filtering options were set to only include loci found in every species (-p 5) and only those occurring in at least 75% of individuals within those species (-r 0.75). The minimum allele frequency was set to 0.05 and only the first SNP per locus was kept resulting in a 1,536 SNP dataset. Species-level structuring of this dataset was evaluated in fastSTRUCTURE v.1.04 with Structure_threader. Models were fitted with the number of genetic clusters (K) set to range from 1 to 10. The most suitable value of K was selected using the chooseK.py function from the fastSTRUCTURE package and visualized using Distruct v.2.3 (Figure 1c). The SNP dataset was then used to reconstruct a maximum likelihood phylogenetic tree of these samples in W-IQ-Tree version 1.6.12. The substitution model used was TVM+F+G4 and branch support was calculated using both 1000 ultrafast bootstrap replications and a Shimodaira–Hasegawa like approximate likelihood-ratio test. Branch support is not shown in Figure 1b, though there was unambiguous support for the grouping of each species.
